# Network-Based Metabolism-Centered Screening of Potential Drug Targets in *Klebsiella pneumoniae* at Genome Scale

**DOI:** 10.3389/fcimb.2019.00447

**Published:** 2020-01-14

**Authors:** Müberra Fatma Cesur, Bushra Siraj, Reaz Uddin, Saliha Durmuş, Tunahan Çakır

**Affiliations:** ^1^Computational Systems Biology Group, Department of Bioengineering, Gebze Technical University, Gebze, Turkey; ^2^Dr. Panjwani Center for Molecular Medicine and Drug Research, International Center for Chemical and Biological Sciences, University of Karachi, Karachi, Pakistan

**Keywords:** *Klebsiella pneumoniae*, infection, genome-scale metabolic networks, pathogen, flux balance analysis, drug target

## Abstract

*Klebsiella pneumoniae* is an opportunistic bacterial pathogen leading to life-threatening nosocomial infections. Emergence of highly resistant strains poses a major challenge in the management of the infections by healthcare-associated *K. pneumoniae* isolates. Thus, despite intensive efforts, the current treatment strategies remain insufficient to eradicate such infections. Failure of the conventional infection-prevention and treatment efforts explicitly indicates the requirement of new therapeutic approaches. This prompted us to systematically analyze the *K. pneumoniae* metabolism to investigate drug targets. Genome-scale metabolic networks (GMNs) facilitating the systematic analysis of the metabolism are promising platforms. Thus, we used a GMN of *K. pneumoniae* MGH 78578 to determine putative targets through gene- and metabolite-centric approaches. To develop more realistic infection models, we performed the bacterial growth simulations within different host-mimicking media, using an improved biomass formation reaction. We selected more suitable targets based on several property-based prioritization procedures. KdsA was identified as the high-ranked putative target satisfying most of the target prioritization criteria specified under the gene-centric approach. Through a structure-based virtual screening protocol, we identified potential KdsA inhibitors. In addition, the metabolite-centric approach extended the drug target list based on synthetic lethality. This revealed the importance of combined metabolic analyses for a better understanding of the metabolism. To our knowledge, this is the first comprehensive effort on the investigation of the *K. pneumoniae* metabolism for drug target prediction through the constraint-based analysis of its GMN in conjunction with several bioinformatic approaches. This study can guide the researchers for the future drug designs by providing initial findings regarding crucial components of the *Klebsiella* metabolism.

## Introduction

*Klebsiella pneumoniae*, originally discovered in the lung samples of pneumonia patients, is a gram-negative, facultative anaerobic bacterium within the Enterobacteriaceae family (Friedlander, 1882). It can inhabit a wide range of environments, and it is also a part of normal flora of human (Bachman et al., 2015; Navon-Venezia et al., 2017). The opportunistic pathogen colonizes in human mucosal surfaces and can spread to other tissues like respiratory tract (Li et al., 2014; Paczosa and Mecsas, 2016). *K. pneumoniae* is among the six pathogens known as “ESKAPE” pathogens, a group of resistant strains that effectively escape from the activity of most of the available antimicrobial drugs (Taneja and Kaur, 2016). A reduction in the antimicrobial activity of many available drugs along with ever-increasing prevalence of the resistant *Klebsiella* strains poses a serious therapeutic challenge. This growing threat affects public health and global economic cost. *Klebsiella* infections primarily affect immunocompromised patients, and they may be treated by the use of β-lactams and other antibiotics (Doorduijn et al., 2016; Paczosa and Mecsas, 2016). On the other hand, even healthy individuals can suffer from the hypervirulent *Klebsiella* infections (e.g., meningitis, pneumonia, endophthalmitis, cellulitis, and pyogenic liver abscesses), and conventional medicine has failed to eradicate such infections (Doorduijn et al., 2016; Paczosa and Mecsas, 2016; Santajit and Indrawattana, 2016). Therefore, identification of novel drugs, use of synergistic drug combinations and drug repositioning remain areas of active investigation (Sun et al., 2016; Taneja and Kaur, 2016), pointing to the crucial role of post-genomic approaches to cope with *Klebsiella* infections (Bachman et al., 2015; Santajit and Indrawattana, 2016). In this context, the evaluation of whole metabolism of the pathogen at genome scale can provide comprehensive insight for the elucidation of more efficient drug targets and enable a deeper understanding of the pathogen phenotype.

Genome-scale metabolic network (GMN) models are commonly used to decipher pathogen and host metabolisms since they offer a systems-wide approach (Durmus et al., 2015). *In silico* analyses based on GMNs can significantly narrow down putative drug targets. Thus, systems biology approach reduces the dependency to labor-intensive, costly and time-consuming experimental approaches. Flux Balance Analysis (FBA) is the most widely used *in silico* analysis method to predict intracellular flux distributions from GMNs at steady-state, which solves an optimization problem satisfying a predefined objective function (e.g., maximal growth rate; Varma and Palsson, 1994; Edwards et al., 2002; Orth et al., 2010). When FBA is used to simulate gene deletion phenotypes, it provides significant quantitative insights about the bacterial metabolism, pathway activities, and potential drug targets (Cesur et al., 2018). To date, this approach has been commonly used in drug target discovery process at systems-level for different pathogens (Raman et al., 2008; Plata et al., 2010; Perumal et al., 2011; Ahn et al., 2014; Larocque et al., 2014; Presta et al., 2017). GMN models are available for different *K. pneumoniae* strains (Liao et al., 2011; Henry et al., 2017; Ramos et al., 2018; Norsigian et al., 2019). The first *K. pneumoniae* model at the genome level, called iYL1228, appeared in 2011 for the MGH 78578 strain (Liao et al., 2011). The authors refined and validated their model by testing the qualitative growth phenotype of the organism on several nutrient sources. They have also identified essential genes in the organism *in silico*, in aerobic minimal medium. The second model was developed for rifampin-resistant highly virulent strain KPPR1 using the GMN of the MGH 78578 strain as a reference (Henry et al., 2017). Similar validation analyses were performed using the model, termed iKp1289, and essential genes were identified *in silico* in aerobic rich media. Both works lack a GMN-based identification of drug target candidates. In a recent work, a GMN of multi drug resistant (MDR) Kp13 strain was developed (Ramos et al., 2018). The authors did not perform an FBA-like analysis based on the calculation of flux distributions. Rather, they used the network topology of the reconstructed GMN to prioritize drug targets in combination with genomic, transcriptomic and structure-based information. More recently, the GMN models of diverse *K. pneumoniae* strains with different levels of antibiotic resistance were reconstructed (Norsigian et al., 2019). They were used to predict the catabolic capabilities of these strains.

Here, we aim to provide a better insight into *K. pneumoniae* metabolism in different host-mimicking conditions in order to reveal putative drug targets through gene- and metabolite-centric approaches. To our knowledge, this is the first comprehensive effort on the investigation of the *K. pneumoniae* metabolism through the constraint-based analysis of its GMN in conjunction with several bioinformatic approaches to reveal the most suitable targets. The subsequent major step in the therapeutic strategies is inhibitor discovery against the most remarkable targets. Structure-based *in silico* drug prediction is a powerful method to explore suitable inhibitors among the available chemical library compounds. In this study, 2-dehydro-3-deoxyphosphooctonate aldolase (KdsA) was reported as the high-ranked putative target satisfying most of the target prioritization criteria specified under the gene-centric approach. Using molecular docking, we attempted to elucidate the interaction dynamics of the KdsA enzyme and to identify possible inhibitors. We identified various potential anti-infectious agents interacting with the enzyme. Taken together, we suggested the putative targets and the KdsA inhibitors through the comprehensive bioinformatic analyses in this work. These findings can provide crucial insights to guide experimentalists for the development of new drugs.

## Materials and Methods

### Metabolic Network Model

The genome-scale metabolic network of *K. pneumoniae* MGH 78578 (GenBank accession number: CP000647), iYL1228 (Liao et al., 2011) accounting for 1,228 genes and 1,658 metabolites involved in 2,262 reactions was used in this work. The GMN covers a set of reactions associated with the metabolism of amino acids, nucleic acids, fatty acids, cofactors, and carbon sources. It contains a reaction for biomass formation to simulate maximal growth conditions. An updated version of the biomass reaction was used in this work (see the next section). The energy required for the biomass formation, termed growth-associated maintenance, was set to 71.7 mmol ATP/gDW/h in the model while the non-growth-associated maintenance, which is the energy dedicated to cellular functions apart from growth (e.g., motility and repair), was set to 6.8 mmol ATP/g dry weight (gDW) (Liao et al., 2011).

### Biomass Reaction

Based on a recent work that drew attention to the importance of inclusion of essential cofactors in biomass formation reactions, we improved the biomass formulation of the original model through integration of universally essential cofactors of prokaryotes [NAD, NADH, NADP, NADPH, FAD, coenzyme A (CoA), flavin mononucleotide (FMN), pyridoxal 5′-phosphate (PYDX5P), and S-adenosyl-L-methionine (SAM/AMET)] (Xavier et al., 2017) to represent the bacterial composition better. In this context, we added each missing cofactor to the biomass formation reaction with a stoichiometric coefficient based on the biomass formula of a GMN model of *Escherichia coli* K-12 MG1655 (Orth et al., 2011). The newly added coefficients with the magnitudes between 10^−3^ and 10^−5^ did not affect the capability of the model to predict the growth rates in different growth conditions (data not shown). In addition, the coefficient of FMN was not available in the *E. coli* model and it was taken as 1 × 10^−5^ in this study. A low coefficient value ensured the necessity of cofactors for growth, without disturbing carbon flows in the metabolic network. The coefficients for the new biomass formation reaction is available in Supplementary Dataset 1.

### Simulation Constraints

Host microenvironment is an important source of the nutrients supporting the bacterial growth. More realistic infection models can be developed by mimicking the host conditions in order to reveal the phenotype of *K. pneumoniae* within the host and to identify novel efficacious antimicrobial drugs. Using FBA, we simulated three different growth conditions: human body fluid (HBF) (Hadi and Marashi, 2014), sputum-macrophage (SM) (Bordbar et al., 2010; Oberhardt et al., 2010), and a more generic host medium. One hundred and twenty of four hundred and five metabolites in the HBF were defined as exchange metabolites in the model, and they were used here to represent HBF medium. Thus, the HBF medium consists of 120 metabolites, and SM medium includes 40 metabolites defined in the *Klebsiella* model. For a more generic host medium, common metabolites in both iYL1228 and Recon 2 (a comprehensive literature-based human metabolic network including 1,789 genes, 7,440 reactions, and 2,626 unique metabolites; Thiele et al., 2013) were identified through the name and ID matching (López-Ibáñez et al., 2016). There are 192 human-matched metabolites in the pathogen metabolic network for which there are defined uptake reactions. These metabolites were used to represent the generic host medium. Compositions of all growth media used in this work are listed in Supplementary Dataset 2. All conditions were mimicked by setting maximum uptake rates of all components within the media in question to 10 mmol gDW^−1^ h ^−1^ in the model during the simulations while the uptake of other compounds were blocked. Subsequently, maximization of the rate of biomass formation reaction was set as the objective in FBA to predict the growth rate.

### Gene-Centric Identification of Drug Targets

Essential genes (EGs) of *K. pneumoniae*, which are indispensable for survival, were determined via *in silico* deletion of each metabolic gene using FBA and the inspection of the corresponding bacterial growth rate. Single-gene knockout studies were performed under specific growth conditions explained above. These perturbations were achieved by setting the rate of the associated reaction(s) to zero for each gene. Compensatory functions of isoenzymes were also considered during the gene deletions. The effect of each single-gene knockout on the biomass production was evaluated based on a cut-off of 1% of the maximum wild-type growth rate (Pratapa et al., 2015). The use of zero as the cut-off led to the same results. Therefore, the genes were considered as essential if their deletions resulted in a substantially reduced growth rate (smaller than the cut-off) relative to the wild-type cell (Supplementary Dataset 3).

Homology analysis is crucial to avoid any undesired harmful effects of the drugs. We identified the essential gene products sharing little to no homology with human proteins. To do so, the essential pathogen proteins were subjected to BLASTp search against human protein sequences in Refseq database (Pruitt et al., 2007) at an expected value (E-value) cut-off of 1 × 10^−4^ (Jamal et al., 2017; Presta et al., 2017). The proteins having <30% sequence identity with their human counterparts were considered as non-homologous (Presta et al., 2017). Hence, a putative drug target list consisting of the non-homologous essential *K. pneumoniae* proteins was compiled. To characterize the putative drug targets, pathways associated with these proteins were identified by means of KOBAS v3.0 web server. It identifies significantly enriched pathways using KEGG and BioCyc pathway databases (Wu J. et al., 2006). We chose a closely related and well-characterized model organism *E. coli* K-12 MG1655 to annotate the putative targets of *K. pneumoniae*. The enriched pathways were determined using a false discovery rate threshold of 10^−4^.

### Prioritization of Putative Drug Targets

Through a systematic workflow, the non-homologous drug target candidates identified by the gene-centric approach were prioritized to discover more effective therapeutic targets. To this aim, *in silico* screening was employed based on subcellular localization, druggability, antibiotic resistance, virulence, and distribution of the target candidates in prominent pathogenic strains (conservation).

#### Subcellular Localization Prediction

Subcellular localization is among the factors determining protein function because compartments include various compounds contributing to the function of a protein. In other words, cellular compartments and protein function are interconnected. Furthermore, localization provides significant information about the nature of the putative targets for drug design. Outer membrane-associated and extracellular proteins may be vaccine targets. On the other hand, small-molecule therapeutics should be designed against cytoplasmic and periplasmic targets (Uddin et al., 2015; Kumar et al., 2016). To address the localization information of the putative drug targets, PSORTb v3.0.2 (scoring cut-off of 7.5; Yu et al., 2010; Ramos et al., 2012; Uddin et al., 2015), CELLO2GO (E-value cut-off of 1 × 10^−4^; Yu et al., 2014), and iLoc-Gneg (Xiao et al., 2011) were used. If the cellular compartment of a protein was confirmed by at least two web servers, it was considered to be correctly predicted. Contradictory results were cross-checked via UniProt database (Wu C. et al., 2006) and a comprehensive study on annotation and localization of *E. coli* K12 proteome by Lopez-Campistrous and colleagues (Lopez-Campistrous et al., 2005).

#### Determination of Druggable Proteins

Druggability is defined as the ability of a target to bind drug-like chemical compounds with high affinity. It is an important criterion in the target prioritization because the proteins with higher druggability are more vulnerable to drugs (Ludin et al., 2012; Shende et al., 2017). DrugBank is a useful knowledgebase including a comprehensive up-to-date data regarding drug-target interactions (Wishart et al., 2008). Druggability assessment was performed using the DrugBank database, to evaluate the risk to invest in a putative target. Sequence search option in the database was used to perform BLASTp with the default parameters. This led to the alignment of the non-homologous proteins of *K. pneumoniae* against the known drug targets in the DrugBank database. Degree of the druggability was determined using the E-value cut-off of 1 × 10^−25^ (Holman et al., 2009; Chawley et al., 2014).

#### Antibiotic Resistance Screening

Antibiotic resistance leads to the emergence of antibiotic-unresponsive pathogens, against which they were previously susceptible. It occurs owing to a widespread use of the antibiotics and results in the counteraction of conventional treatment approaches (Kumar et al., 2011; Beceiro et al., 2013). Transfer of the resistance-conferring plasmids promotes dissemination of the MDR isolates within the Enterobacteriaceae family members (Kumar et al., 2011). Development and dissemination of the resistance led us to investigate existing or putative antibiotic resistance genes (ARGs) within the identified putative drug target lists. Antibiotic resistance screening was performed using ARG-ANNOT tool, which harbors up-to-date information on the ARGs against 11 antibiotic classes (Gupta et al., 2014; Jia et al., 2017). In this work, a recently updated data (May, 2018) in ARG-ANNOT was used through BioEdit software (Gupta et al., 2014) with an E-value cut-off of 1 × 10^−4^ and 65% sequence identity.

#### Identification of Potential Virulence Factors

There is an interplay between antibiotic resistance and virulence. Virulence refers to the degree of pathogenicity, the capacity of a pathogen to cause disease, which has some common characteristics with the antibiotic resistance. Both can disseminate via the gene transfer among bacteria, and they support survival of the pathogens within the harsh host environment by enhancing the defense against the host immune response and antimicrobials. To cope with the host, they have some common mechanisms such as modification of the cell wall, use of some global transcriptional regulators, production of efflux pumps and porins (Trevor and Snow, 2005; Beceiro et al., 2013; Llobet et al., 2015). Particularly, combinatorial therapies including application of anti-virulence agents and antibiotics may promote more effective drug therapy and reduce antibiotic resistance development (Beceiro et al., 2013). Thus, we concentrated on the investigation of potential virulence factors (VFs) in addition to ARGs. To elucidate VFs within the identified potential drug target lists, core dataset within the VFDB database was used (Chen et al., 2016). This database includes up-to-date data related to VFs of 30 prominent bacterial pathogens. We annotated putative virulence factors using BLASTp option in the database with the E-value cut-off of 1 × 10^−4^, bit score of 100 and identity threshold of 65% (Gawade and Ghosh, 2018).

#### Broad-Spectrum Analysis

A homology analysis against diverse infectious bacteria was performed to evaluate the broad distribution of the candidate targets. This analysis may provide a clinically effective opportunity for the treatment of co-infections or multiple infections. In addition, the conservation of a gene may indicate a lower level of mutation rate, so targeting such genes may delay the development of antibiotic resistance. Here, broad-spectrum analysis was performed through PBIT web browser (Shende et al., 2017). To this aim, we executed the BLASTp search in the tool, which performs a similarity search against protein sequences of 181 pathogenic organisms with the E-value cut-off of 1 × 10^−5^, bit score of 100 and sequence identity of 35%. If a protein was identified to be common in at least 40 diverse pathogenic strains, it was considered as a broad-spectrum target (Mondal et al., 2015).

### Structure-Based Inhibitor Discovery

The *K. pneumoniae* MGH 78578 KdsA enzyme was determined as the top candidate drug target through the target prioritization step. The amino acid sequence of the enzyme was subjected to BLAST search against the Protein Data Bank (PDB) to find its crystal structure. The sequence identity and similarity from multiple sequence alignments were further confirmed by ClustalW (Thompson et al., 2003). The crystal structure of the matched *E. coli* enzyme (PDB code: 1D9E) was prepared as a receptor via UCSF chimera to perform the structure based analyses (Pettersen et al., 2004). The active site residues and corresponding cavity volumes for 1D9E were assigned based on the reported literature (Radaev et al., 2000). In order to perform a structure-based virtual screening protocol, a subset of the ligand database Aldrich was retrieved from the ZINC database, containing 18,142 compounds in ready-to-dock, 3D format (Irwin et al., 2012). To perform the docking experiment, the program AutoDock Vina was used (Trott and Olson, 2010). The docking program Vina requires input files in PDB format. A grid box was set with x, y, and z dimensions of 140 Å. Vina generated maximum nine poses of each compound interacting with the protein. The docked complexes with top ranked Vina scores were used to narrow down the compounds based on ranking scores.

### Metabolite-Centric Identification of Drug Targets

Essential metabolites (EMs) of *K. pneumoniae* are among the major components of the bacterial metabolism. Metabolite-centric gene deletion is equivalent to simultaneous removal of all outgoing reactions around each metabolite by constraining the rates of related reactions to zero and the inspection of the corresponding bacterial growth rate. Same as in the gene-centric approach, we employed a cut-off of 1% of the maximum wild-type growth rate (Pratapa et al., 2015) to decide whether a metabolite is essential or not. The simulations were performed in SM, HBF, and generic host media separately. If the blocking of a metabolite resulted in a significant growth attenuation, it was considered as essential. The multiple genes involved in the outgoing reactions of an EM are called synthetic lethal. That is, not individual but simultaneous targeting of their enzymes with an EM-like drug will cause cell death.

Identification of the EMs was followed by a similar filtering method of Kim et al. (2010) to narrow down the metabolite list and to elucidate the enzymatic drug targets. To filter the EMs, we followed two main steps: In the first step, the EMs were screened in terms of their presence in human metabolism for their removal. To this aim, the common metabolites of the *K. pneumoniae* and human were compiled using the human genome-scale metabolic reconstruction, Recon 2 (Thiele et al., 2013), and HumanCyc database (Trupp et al., 2010). This step also ensured the removal of currency metabolites (e.g., ATP, H_2_O, NAD) known as the metabolites involved in many reactions in a metabolic network (Samal and Martin, 2011). The common compounds were detected through the name and ID comparisons (López-Ibáñez et al., 2016) and eliminated to avoid any potential damages to the host metabolism. Hence, the EMs specifically dedicated to the pathogen metabolism were obtained by applying this step. In the second step, the pathogen-specific EMs associated with any human homologous enzymes as substrates or products were removed to avoid possible side effects. To this aim, similar to the analysis applied in gene-centric identification of drug targets, the associated enzymes were evaluated in terms of homology. This was performed using BLASTp search against the human protein sequences in Refseq database (Pruitt et al., 2007), using the same E-value and sequence identity cut-offs as in the gene-centric identification part (1 × 10^−4^ and 30%, respectively). The enzymes in the outgoing reactions of EMs that passed the cut-offs were suggested as the putative drug targets.

## Results and Discussion

Here, we simulated the *K. pneumoniae* MGH 78578 metabolism through the GMN of iYL1228 for the computational prediction of effective drug targets. The available infection models are predominantly based on the simulation of the bacterial growth in laboratory media. Although these conditions are also informative, they are insufficient to represent the host-cell nutrient environment. Therefore, we employed growth simulations in different host-mimicking conditions including SM, HBF, and generic host media.

Sputum is mainly composed of inflammatory components, the lower airway mucus, and bacterial products. It is modified in the inflamed lungs (Turner et al., 2015). *K. pneumoniae* induces production of thick jelly sputum (Fukuyama et al., 2014; Paczosa and Mecsas, 2016). It was also demonstrated that *K. pneumoniae* (considered as an extracellular pathogen) could survive inside alveolar macrophages by blocking phagosome maturation (Cano et al., 2015). Taken together, both host environments including sputum (Oberhardt et al., 2010) and alveolar macrophage (Bordbar et al., 2010) were combined in order to provide novel insight into the pathogenesis and fitness requirements of *K. pneumoniae* within this special medium (i.e., SM medium). It is used to discover the putative drug targets. In addition to sputum, body fluid cultures (e.g., blood, urine) are frequently used for the detection of *K. pneumoniae* (Goroll and Mulley, 2009). Here, another growth medium mimicking the body fluids (i.e., HBF medium; Hadi and Marashi, 2014) and a more generic host medium were also integrated to the GMN for more comprehensive overview of the host environment. In addition to the simulation of more realistic growth conditions, improving the biomass formation reaction is also crucial to ensure more accurate representation of the bacterial metabolism. Furthermore, model-predicted knockout phenotypes are significantly affected by the biomass reaction. Therefore, the biomass formulation of the original model was improved via the integration of the universally essential organic cofactors involved in the biomass compositions of prokaryotes (Xavier et al., 2017).

**Figure 1:**
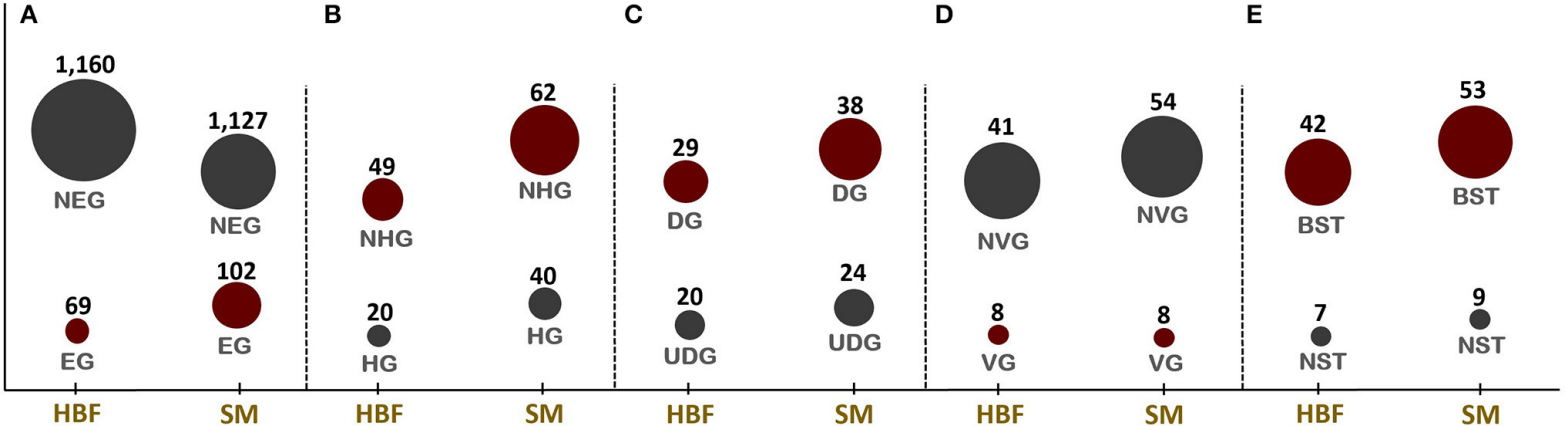
Numbers of candidate drug targets predicted through in silico gene essentiality simulations in host-mimicking medium [i.e., human body fluid (HBF) and sputum-macrophage (SM)] are given in **(A)**. The genes predicted by the HBF simulation are the subsets of SM derived results. Numbers of human non-homologous genes among essential genes are given in **(B)** while **(C–E)**, respectively, give the results of the prioritization applied to the essential non-homologous genes using druggability, virulence factor, and broad-spectrum analyses. NEG, non-essential gene; EG, essential gene; NHG, non-homologous gene; HG, homologous gene; UDG, undruggable gene; DG, druggable gene; NVG, non-virulence gene; VG, virulence gene; NST, narrow-spectrum target; and BST, broad-spectrum target.

The improved simulation conditions provided identification of the putative drug targets in *K. pneumoniae* using the GMN of iYL1228 through both gene- and metabolite-centric approaches. The putative targets predicted through the gene-centric approach were prioritized based on several property-based filtering procedures detailed in the section Materials and Methods (Figures 1A–E). The compounds interacting with the top target (KdsA) among the virulent, druggable, and broad-spectrum enzymes were predicted using a structure-based virtual screening. Accordingly, the active site of the enzyme was explored and docking was employed to shed light on the interaction dynamics and putative inhibitors of this enzyme.

### Gene-Centric Approach

#### Identification of Potential Drug Targets

Identification of EGs is the first key-step in most drug discovery pipelines. This step can be employed through experimental or computational methods. Here, the GMN was constrained by imposing the condition-specific nutrients. The EGs were defined by a significant reduction in the bacterial growth rate based on *in silico* knockout of each gene. A total of 69 and 102 EGs were predicted in the metabolic simulations in HBF and SM growth media, respectively (Figure 1A). The EGs identified via SM simulation cover all 69 EGs identified in the HBF simulation (Supplementary Dataset 3). The gene essentiality analysis for the generic host medium resulted in the identification of 67 essential genes, which are the subsets of the HBF- and SM-derived results. The remaining SM-specific EGs were defined as “conditionally essential” due to their indispensability for the growth in only SM medium.

When compared to the essential genes identified by Bachman et al. (2015), a small fraction of the predicted EGs was found to be common. Using a mouse model of pneumonia, they identified 69 mutants with over 10-fold fitness defect and 333 mutants with an over two-fold fitness defect for *K. pneumoniae* KPPR1. They next investigated the conservation of these genes in several *K. pneumoniae* strains. A comparison with *K. pneumoniae* MGH78578 genome revealed that many of them are shared by both strains (Bachman et al., 2015). Almost half of these genes were found in iYL1228. The EGs predicted in the current work may have lost their essentiality during mouse infection. This highlights the discriminative role of the growth condition on the gene essentiality. Nonetheless, we proceeded with all EG sets for a more comprehensive analysis, without making an additional filtering based on the growth condition.

**Figure 2:**
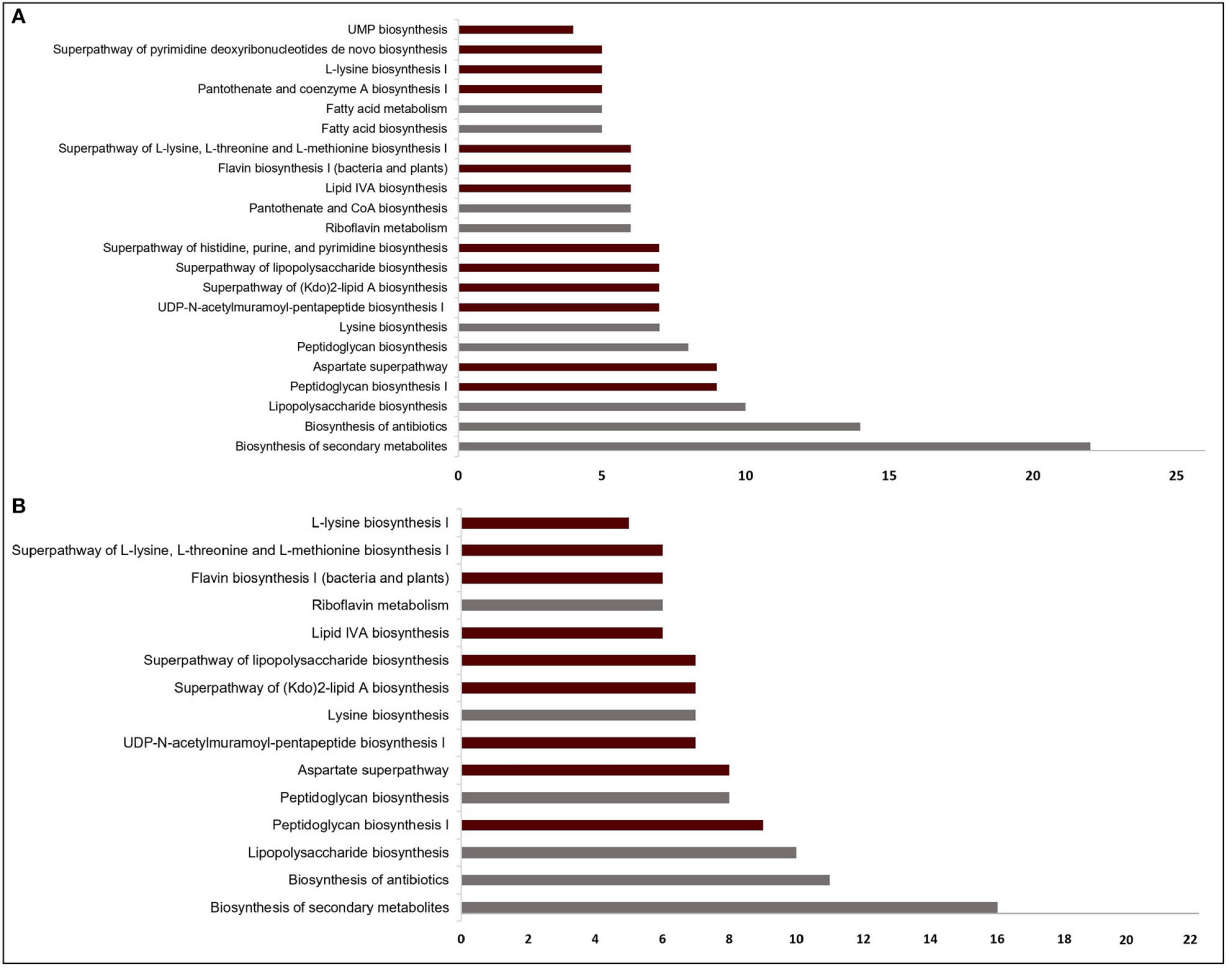
Significantly enriched pathways (FDR < 10^−4^) regarding the targets from **(A)** sputum-macrophage (SM) and **(B)** human body fluid (HBF) simulations. The pathways that appear specifically in SM medium **(A)** point to the common functions of SM-specific conditionally essential genes. Only a subset of the pathways in **(A)** covers all pathways identified for the HBF medium **(B)**. For each pathway, the bar shows number of putative targets involved in that pathway. The pathways from KEGG pathway database is given by gray bars while the bars representing BioCyc pathways are highlighted in claret red.

The EGs were evaluated in terms of homology with human proteome to avoid off-target effects. The pathogen proteins showing sequence similarity higher than the selected cut-off were discarded from the list of putative drug targets. Out of 69 essential proteins predicted by the HBF simulation, 49 were found to be non-homologous to the host proteins. Use of the generic host medium led to a similar result and 49 HBF-derived non-homologous genes were found to cover the 47 non-homologous genes identified via the generic host medium. The two genes not predicted as essential in the generic host medium compared to HBF-based medium are *nadD* and *nadE*. Indeed, there are many works supporting their potentials as drug targets in diverse bacterial pathogens and investigation of possible inhibitors against them (Sorci et al., 2009; Huang et al., 2010; Rodionova et al., 2014; Wang et al., 2017). This highlighted the power of HBF with 120 metabolites compared to the generic host medium with 192 metabolites to properly simulate the host environment. The remaining analyses were performed for this more specific but powerful host condition by ignoring the generic host medium. Only one of the aforementioned genes (*nadE*) is available in the prioritized gene list given in Table 1 (see the footnote to the table). In the same manner, we performed homology analysis for 102 EGs from the SM simulation, out of which only 62 were identified as non-homologous proteins (Figure 1B). The SM-specific collection of 62 genes includes all non-homologous EGs from the HBF simulation. The identified non-homologous genes were shortlisted as potential drug targets. To characterize the candidate targets, we first used pathway enrichment analysis. In this regard, we investigated metabolic pathways in KEGG and BioCyc databases associated with the putative targets. The potential targets from the SM simulation were found to be mainly involved in the metabolism of the bacterial membrane structure, amino acid biosynthesis, and cofactor production (Figure 2A). Several pathways were specifically identified only for the SM simulation, which also corresponds to the common functions of the conditionally essential genes required for survival in the SM media. The conditionally essential genes were found to be predominantly involved in nucleotide and cofactor biosynthetic processes. Among these, *panBCD* gene cluster is crucial to manage pantothenate synthesis pathway, which is crucial for CoA production (Leonardi and Jackowski, 2007). The pantothenate synthesis may be a promising target considering that the CoA participates in many vital metabolic processes such as degradation and synthesis of the fatty acids, production of non-ribosomal proteins, and biosynthesis of phospholipids (Leonardi and Jackowski, 2007; Spry et al., 2008). It is worth emphasizing that essentiality of the *panBCD* is dependent on the intrinsic capacity of the microorganisms to import exogenous pantothenate (Gerdes et al., 2002). *K. pneumoniae* MGH 78578 has a sodium/panthothenate symporter (encoded by *panF* gene) dedicated to the transportation of pantothenate. Since HBF medium includes pantothenate unlike SM medium (Supplementary Dataset 2), the genes in the *panBCD* gene cluster were predicted as essential in only SM simulation. This also shows that medium selection greatly influences the predictive accuracy. On the other hand, any HBF-specific cellular processes could not be identified since HBF-based non-homologous EGs are the subset of SM-based EGs (Figure 2B).

**Table 1:** Prioritization of putative drug targets (elucidated via gene essentiality and homology analyses) in terms of druggability, virulence, and broad-spectrum distribution.

**Putative drug target list**		**Prioritization of putative drug targets**		
**Locus ID**	**Description**	**Virulence analysis**	**Druggability analysis**	**Broad spectrum analysis**
KPN_02230	kdsA	+	12	107
KPN_00194	lpxA	+	2	107
KPN_00100	lpxC	+	6	101
KPN_00236	gmhA	+	1	107
KPN_03963	hldD	+	2	58
KPN_01284	*fabI*	–	27	117
KPN_00478	*purE*	–	1	154
KPN_02000	ribC	–	1	95
KPN_00020	ribF	–	3	94
KPN_00367	ribH	–	14	128
KPN_03599	murA	–	7	200
KPN_04350	murB	–	2	56
KPN_00095	murC	–	3	100
KPN_00092	murD	–	8	59
KPN_00089	murE	–	3	110
KPN_00090	murF	–	1	69
KPN_00094	murG	–	1	78
KPN_04256	murI	–	3	61
KPN_04135	glmU	–	5	149
KPN_04352	coaA	–	3	59
KPN_03974	coaD	–	5	148
KPN_00141	*panB*	–	2	138
KPN_00140	*panC*	–	5	130
KPN_00139	*panD*	–	2	94
KPN_03979	dfp	–	2	151
KPN_02812	dapA	–	1	139
KPN_00039	dapB	–	2	96
KPN_00179	dapD	–	5	67
KPN_01096	tmk	–	1	97
KPN_01074	*pyrC*	–	4	59
KPN_03983	*pyrE*	–	2	53
KPN_01277	*pyrF*	–	3	92
KPN_01228	$nadE^{*}$	–	6	78
KPN_03799	asd	–	3	63
KPN_02202	galU	+	–	208
KPN_00192	lpxD	+	–	74
KPN_02493	ugd	+	4	30

*Targets predicted by at least two prioritization approaches are given here. The genes that satisfy all three criteria are given at the top of the table. Target candidates from human body fluid simulation (HBF) are the subset of sputum-macrophage (SM) simulation-derived results and they are given in red letters. The number of the bacterial pathogen genomes containing the potential targets and the number of interacting drugs are listed in the table*.

^*^All HBF medium-predicted genes in the table were also predicted to be drug targets via the generic host medium simulation, apart from nadE. The nadE gene was classified as essential by HBF and SM simulations, but it was not predicted by the generic host medium simulation.

#### Prioritization of Potential Drug Targets

**Figure 3:**
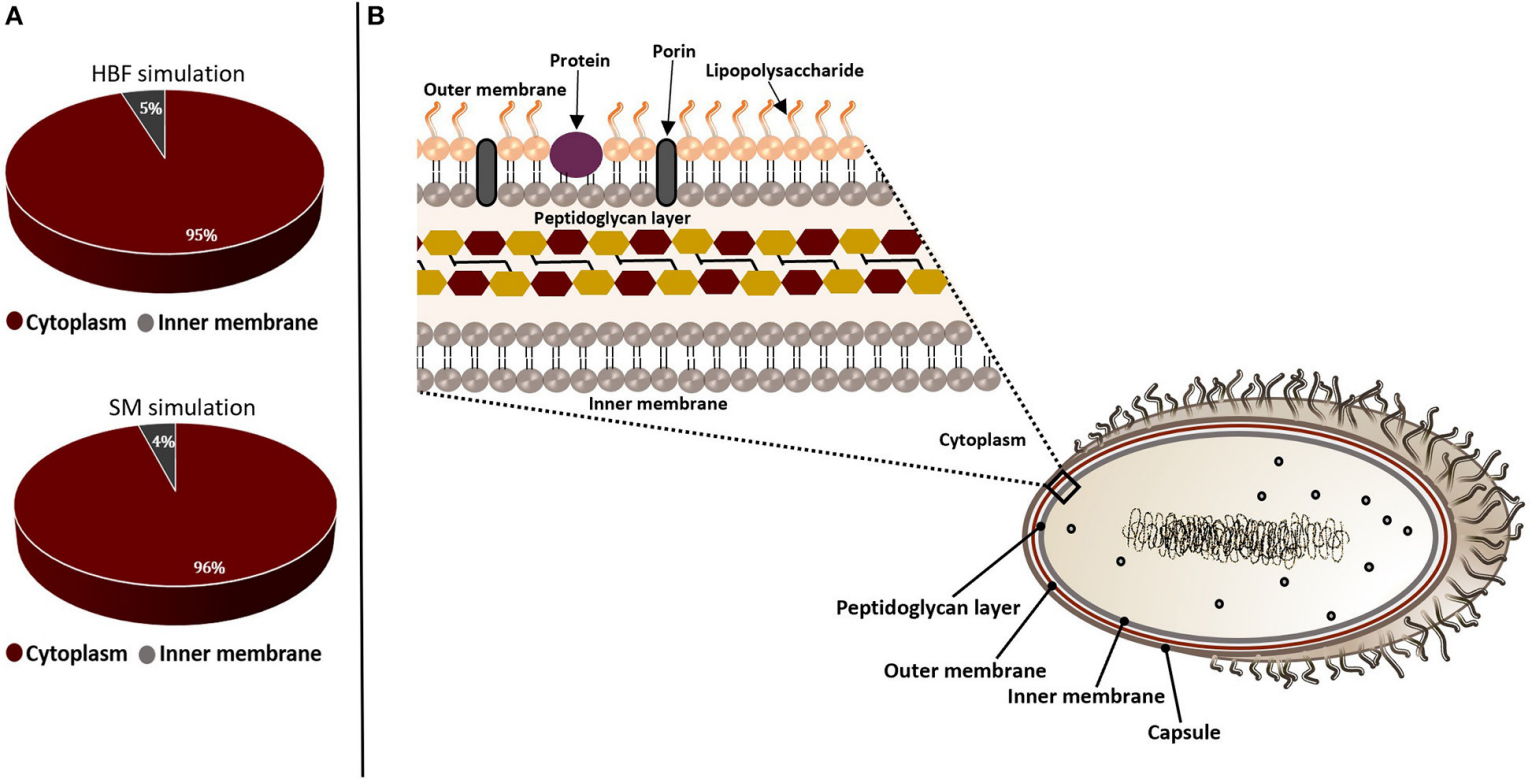
Subcellular localizations of the putative drug targets from gene essentiality and homology analyses. The candidate genes identified by sputum-macrophage (SM) simulation are a subset of genes predicted through the human body fluid (HBF) simulation. **(A)** The pie-charts demonstrate the distribution of the drug targets in *K. pneumoniae* from human body fluid (HBF) and sputum-macrophage (SM) simulations on the basis of their subcellular localizations. **(B)** A Gram-negative bacterial cell wall consists of an outer membrane and peptidoglycan layer. The peptidoglycan layer is located within periplasmic space between outer and inner membranes. The outer membrane is composed of lipopolysaccharides, porins and receptor proteins.

The non-homologous essential pathogen proteins were further characterized to select a set of targets by assessing suitability of these candidates. In this regard, they were filtered through the target prioritization pipeline. Subcellular localization was used as the first criterion since the localization information is crucial to provide an insight into structural and functional characteristics of the proteins. Most of the putative targets were found to be cytoplasmic while a small amount localizes at inner membrane, as illustrated in Figure 3. This result indicates that our list does not include any vaccine targets. Thus, small molecule drug design is required to allow entry into the bacterial cells and inhibit these targets.

The ability of proteins to bind to drug-like molecules is as much important as protein localization in the drug design process. This is because not all protein structures are capable of binding to drug-like compounds (Shende et al., 2017). Therefore, we also assessed the putative drug targets in terms of druggability. Thirty-eight targets from the SM simulation were found to be druggable (Figure 1C). They play key roles in the synthesis of cell wall (i.e., outer membrane and peptidoglycan layer), cofactor formation, nucleotide metabolism and amino acid biosynthesis. Among them, 11 candidates showed affinity with at least five drug molecules (Table 1). We next investigated the significantly matched approved drugs interacting with the 38 putative targets. Gentamicin, pyrophosphoric acid, ethionamide, isoniazid, and fosfomycin approved by the Food and Drug Administration (FDA) were found to interact with three putative targets [NAD^+^ synthetase (NadE), enoyl-(acyl carrier protein) reductase (FabI), and UDP-N-acetylglucosamine 1-carboxyvinyltransferase (MurA)]. Druggability analysis identified 29 targets from HBF simulations as druggable (Figure 1C), all of which were members of 38 target-set identified via SM simulations. A complete list of drugs is presented in Supplementary Dataset 4.

**Figure 4:**
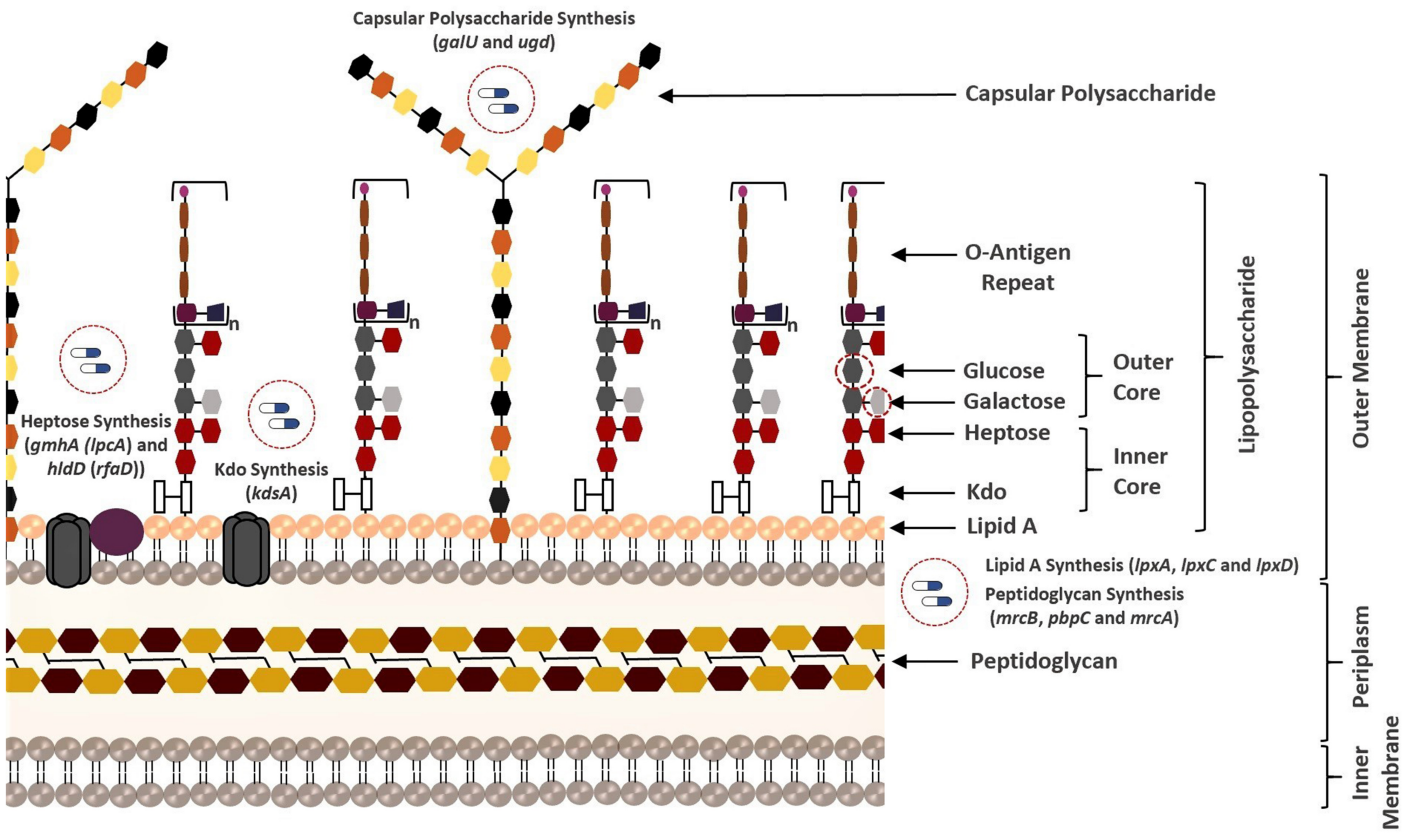
Overview of all bacterial membrane-associated drug targets suggested in this study. The membrane structure was illustrated based on the well-known membrane compositions of the *Escherichia coli*. The putative targets responsible for biosynthesis of the membrane components of *K. pneumoniae* MGH 78578 are highlighted.

The resistance that emerges through the transfer of ARGs and/or mutations requires an urgent introduction of efficient therapeutic strategies. This is because the acquisition of resistance genes promotes predomination of resistant populations via the elimination of wild-type microorganisms (Munita and Arias, 2016). Here, the antibiotic resistance screening was employed as another important prioritization step to improve the success in the eradication of the *Klebsiella* infections. In this regard, the presence of any resistance-associated proteins within the putative drug target list was investigated. However, no ARGs could be determined. This result prompted us to focus on VF screening. There is an obvious relationship between VFs (e.g., capsular polysaccharide (CPS), lipopolysaccharide (LPS), fimbriae, outer membrane proteins, and siderophores) and ARGs considering that they share some common characteristics (Thornley and Horne, 1962; Beceiro et al., 2013; Paczosa and Mecsas, 2016). Thus, targeting the VFs may significantly damage the pathogen by reducing both its pathogenicity and drug resistance. As mentioned in the section Materials and Methods, we used VFDB to reveal VFs within the putative drug target list. Regardless of simulation conditions (i.e., HBF or SM), eight virulence genes including *lpxA, lpxC, lpxD, ugd, hldD* (formerly *rfaD*), *galU, kdsA*, and *gmhA* (formerly *lpcA*) were predicted (Figure 1D). They are primarily involved in the formation of the bacterial membrane (Figure 4).

LPS (also known as endotoxin) is one of the major components of the outer membrane in gram-negative bacteria, and it contributes to protection against antimicrobial molecules in addition to membrane integrity (Deacon et al., 2000). It consists of three principal layers including O-antigen, core oligosaccharide, and lipid A (Maldonado et al., 2016). The core oligosaccharide region of LPS contains inner core [association of 3-deoxy-D-manno-oct-2-ulosonic acid (Kdo) and heptose residues] and outer core [association of hexoses (galactose and glucose) and hexosamines]. Several structural features of the LPS differ within bacterial species. For instance, the Kdo of *K. pneumoniae* LPS is also localized in the outer core unlike *E. coli* (Regué et al., 2005). Phosphoheptose isomerase (encoded by *gmhA*) and ADP-L-glycero-D-manno-heptose-6-epimerase (encoded by *hldD*) catalyze the biosynthesis of the heptose precursors. On the other hand, 2-dehydro-3-deoxyphosphooctonate aldolase (encoded by *kdsA*) is responsible for the synthesis of the Kdo, which links lipid A and core oligosaccharides (Strohmaier et al., 1995). Lipid A is the innermost leaflet of the outer membrane. It supports the integrity of the outer membrane along with the core oligosaccharide, and it is essential for bacterial survival (Barb and Zhou, 2008; Llobet et al., 2015). UDP-N-acetylglucosamine acyltransferase (encoded by *lpxA*), UDP-3-O-acyl-N-acetylglucosamine deacetylase (encoded by *lpxC*), and UDP-3-O-(3-hydroxymyristoyl) glucosamine N-acyltransferase (encoded by *lpxD*) are involved in the lipid A biosynthesis (Clements et al., 2002). Other VFs reported as the putative drug targets in this study play an important role in both LPS and CPS biosynthesis. UDP-glucose pyrophosphorylase (encoded by *galU*) is responsible for the regulation of the intracellular UDP-galactose and UDP-glucose concentrations (Lai et al., 2001; Shu et al., 2009). Conversion of UDP-glucose into UDP-glucuronic acid is managed by UDP-glucose 6-dehydrogenase (encoded by *ugd* at the 3′ end of *K. pneumoniae cps* gene clusters) and this compound is used as a substrate for synthesis of different surface structures (Shu et al., 2009; Mainprize et al., 2013; Figure 4).

To delay the resistance development, another prominent approach is inhibition of the conserved targets. Discovery of more conserved, broadly distributed targets may not only enable to slow resistance development but also facilitate to reveal the targets for a bacterial co-infection or multiple infections. Therefore, we explored the putative targets, homologs of which are widely distributed among pathogens. The analysis resulted in 53 potential broad-spectrum targets from the SM simulation, which are involved in at least 40 strains of the serious pathogens (e.g., *Bacillus cereus, Clostridium botulinum, Acinetobacter baumannii, Haemophilus influenzae, Helicobacter pylori, Mycobacterium tuberculosis, Salmonella typhimurium*, and *Staphylococcus aureus*, among others; Figure 1E). Twenty-two of the targets are distributed across at least 100 strains, and these proteins are mainly responsible for the membrane synthesis or cofactor production. Broad-spectrum analysis of 49 non-homologous essential targets from the HBF simulation revealed 42 possible broad-spectrum antibacterial targets, which are also included in the set of the broad-spectrum targets from the SM analysis (Figure 1E). Eighteen of them are present in at least 100 strains. Overall, the broad conservation of the targets predicted in the study can have a great advantage in avoiding emergence of drug resistance in different pathogenic strains.

#### Analysis of the Prioritized Drug Targets

In-depth examination of the prioritized list of the drug targets reveals a total of 57 non-homologous genes that satisfy at least one prioritization criteria. In a recent multi-omics study by Ramos and colleagues, 29 potential drug targets for MDR *K. pneumoniae* Kp13 were proposed (Ramos et al., 2018). Comparison of the computational predictions revealed 18 common targets with that study.

The genes identified by at least two prioritization approaches are listed in Table 1, which amount to 37 genes. Only five proteins (encoded by *lpxA, lpxC, hldD, gmhA*, and *kdsA*) in this prioritized target list were identified by the three prioritization approaches. That is, those proteins were predicted to be druggable, broad-spectrum, and virulence factors (Table 1). They are responsible for the biosynthesis of the bacterial cell wall components as described before (Figure 4). Of these five targets, LpxA and LpxC were also suggested as putative drug targets by Ramos et al. (2016, 2018) confirming the involvement of these enzymes in polymyxin B resistance. The LpxA exhibits no significant structural or sequence homology with mammalian enzymes (Barb and Zhou, 2008; Joo, 2015). To date, crystal structures of this protein have been revealed for various bacteria such as *E. coli* (Raetz and Roderick, 1995; Williams and Raetz, 2007), and *Leptospira interrogans* (Robins et al., 2009). More recently, the crystal structure of *Moraxella catarrhalis* LpxA was determined and potential inhibitors were suggested by presuming that they may also interact with the LpxAs from other gram-negative bacteria (Pratap et al., 2017). When compared to the LpxA, there is a further effort for the development of the LpxC inhibitors (Lee et al., 2011; Kalinin and Holl, 2017). Considering conservation and essentiality of the LpxC protein among the gram-negative bacteria, it is a quite promising drug target (Barb and Zhou, 2008). In a recent study, 3D structure of MDR *K. pneumoniae* HS11286 LpxC was modeled using X-ray crystallographic structure of *E. coli* LpxC as the template. Based on the molecular docking and molecular dynamics simulations, a uridine-based receptor antagonist was suggested to be a potential inhibitor destabilizing the substrate-binding site of the *K. pneumoniae* HS11286 LpxC (Ahmad et al., 2018a).

In addition to the components in the lipid A synthesis pathway, other enzymes responsible for LPS synthesis are also attractive target candidates (Figure 4). HldD and GmhA are two other proteins predicted as drug targets by the three prioritization approaches, which are crucial for resistance within harsh host environment and against medical interventions. They are especially promising in the design of broad-spectrum antibiotic adjuvants in order to enhance the sensitivity of the pathogens against available antimicrobial agents (Taylor et al., 2008). The HldD, with no mammalian counterpart, is involved in the generation of an obligatory component of the LPS core domain in the most gram-negative bacteria (Deacon et al., 2000; Kuo et al., 2016). A reduced ability of *hldD* mutants (i.e., core-defective mutants) to survive in the host (Deacon et al., 2000) and the contribution of the HldD to the bacterial virulence (Kuo et al., 2016) have been reported. Thus, development of potent epimerase inhibitors may facilitate powerful antibiotic adjuvant therapies. The GmhA is another conserved enzyme. The cell wall-damaging agents are well-tolerated thanks to this enzyme by many gram-negative pathogens [e.g., *Fusobacterium nucleatum* (Kumar et al., 2016), *Neisseria gonorrhoeae* (Wierzbicki et al., 2017), *L. interrogans* (Umamaheswari et al., 2010), and *E. coli* (Taylor et al., 2008)]. Hence, design of the GmhA inhibitors holds promise to cope with the ongoing resistance problem by increasing the antibiotic susceptibility of the pathogens. Homology models and crystallographic structures of this enzyme were investigated for various pathogens to further shed light on structure/function relationship of the enzyme and facilitate the design of alternative therapeutic approaches (Taylor et al., 2008; Umamaheswari et al., 2010). The homology model of *L. interrogans* GmhA provided prediction of 14 novel competitive inhibitors (Umamaheswari et al., 2010).

Based on the prioritization criteria discussed above, the KdsA was defined as the highest-ranked drug target among the target candidates satisfying all criteria defined for the virulence, druggability, and broad-spectrum analyses (Table 1). It is the top candidate since, among the five mentioned proteins, it has the highest number of interacting drugs. In addition, it shares the highest broad-spectrum score with two others. This enzyme may be a promising target, considering that inhibition of the Kdo biosynthesis results in suppression of replication and so cell growth arrest (Xu et al., 2003; Ahmad et al., 2019). The KdsA from different pathogens has been so far proposed as the putative drug targets through *in silico* studies such as metabolic pathway analysis (Perumal et al., 2007; Rath et al., 2016), multi-omics approach (Ramos et al., 2018), subtractive genomics (Amineni et al., 2010), and subtractive proteomics (Ahmad et al., 2018b) as well as through *in vitro* studies (Perumal et al., 2010). Moreover, several Kdo inhibitors with a limited *in vivo* activity, despite their promising *in vitro* applications, have been developed (Du et al., 1999; Birck et al., 2000; Grison et al., 2005; Le Calvez et al., 2009; Harrison et al., 2012; Smyth and Marchant, 2013). These inhibitors were reported to be useful for inhibition of the KdsA activity in different bacteria. More recently, KDO8P oxime was evaluated in terms of the inhibitory capacity, which supported a greater understanding of the binding kinetics. This may lead to more efficient KdsA inhibitors (Gama et al., 2018). Identification of the KdsA protein at the top of the ranked putative target list in the current study and studies on KdsA inhibitors encouraged us to investigate *K. pneumoniae* KdsA inhibitors. We investigated potent inhibitors for this protein through the structure-based virtual screening protocol (see the next section).

It is important to note that we discussed only targets meeting all specified criteria (apart from antibiotic resistance screening) in the target prioritization process. However, the targets identified by most of the prioritization approaches may be also attractive. For instance, Ugd is a promising drug target though it was not prioritized in the broad-spectrum analysis. It is crucial for the biosynthesis of both CPS and LPS (Figure 4), which support gram-positive and gram-negative bacterial evasion of host innate immune response (Mainprize et al., 2013). The correlation between the enzymatic activity of the Ugd and antibiotic resistance indicates convenience of this enzyme as drug target in several pathogens [e.g., *K. pneumoniae* CG43 (Cheng et al., 2010) and *Pseudomonas aeruginosa* (Hung et al., 2007)]. Furthermore, Chen and colleagues demonstrated the inhibitory role of the UDP-glucuronic acid on this enzyme. They identified conformational changes in the enzyme via both competitive and allosteric inhibition along with the increased UDP-glucuronic acid concentration in *K. pneumoniae* NTUH-K2044. Thus, it was highlighted that regulation mechanism of the UDP-glucuronic acid would be guiding in the design of inhibitors (Chen et al., 2011). Another example is GalU, and it was found that *galU*-deficient mutant of *K. pneumoniae* CG43 had a defect in both the utilization of galactose and production of capsular polysaccharide (Figure 4). Hence, *galU* mutation impairs the virulence *in vivo* (Chang et al., 1996).

### Identification of Potential Drugs for KdsA

**Figure 5:**
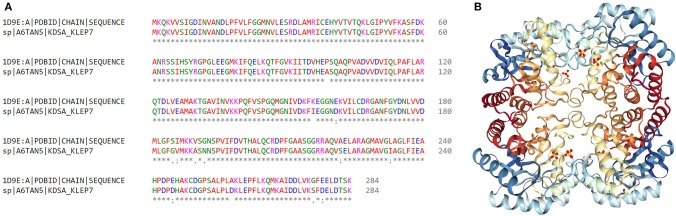
Identification of the most convenient KdsA homolog to investigate the inhibitors against *K. pneumoniae* MGH 78578 KdsA. **(A)** Sequence alignment between the KDO8P synthase enzymes of *E. coli* and *K. pneumoniae*. **(B)** Crystal structure of the *E. coli* KDO8P synthase (PDB code: 1D9E) is represented.

**Figure 6:**
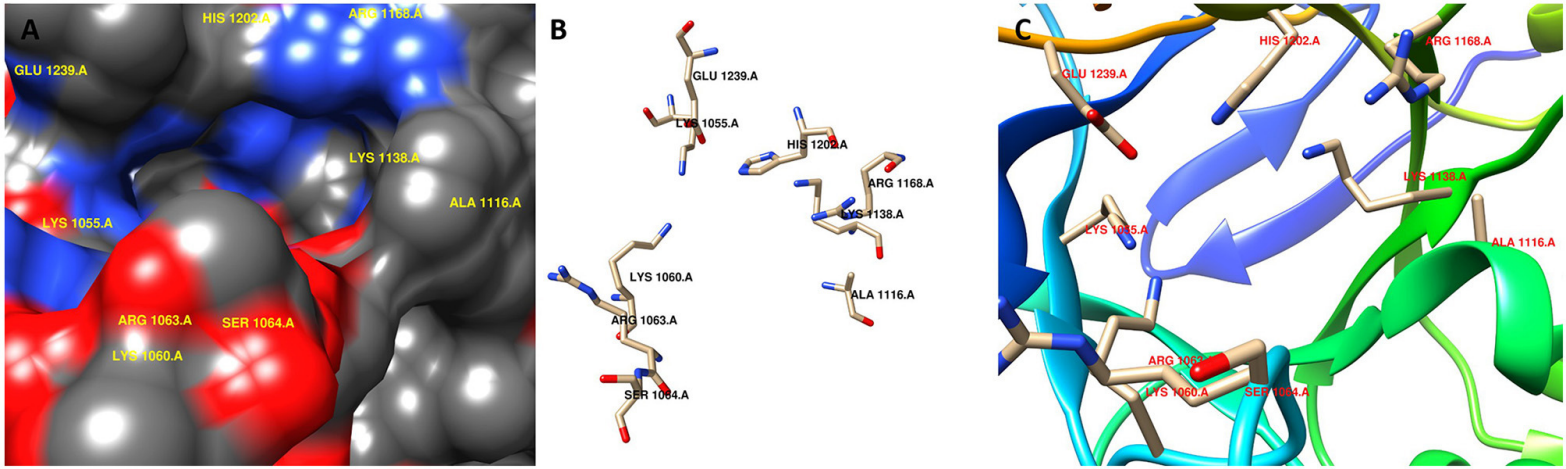
Active site region of the receptor (PDB code: 1D9E). **(A)** surface representation, **(B)** stick representation, and **(C)** ribbon representation.

Computer-aided drug design is a useful approach to reduce time and cost. Therefore, we investigated putative inhibitors against the high-ranked potential target (KdsA) using *in silico* techniques. To our knowledge, this is the first attempt for the *K. pneumoniae* KdsA enzyme. A comparative structure prediction method was firstly used since the 3D structure of *K. pneumoniae* KdsA enzyme was not available. Upon similarity search via BLAST, the 3D structure of KDO8P synthase from *E. coli* was selected (PDB code: 1D9E). The *E. coli* enzyme showed 95.42% sequence identity, 100% query coverage, and had a high resolution with 2.4 Å. Figure 5A shows the sequence similarity between the two proteins. The PDB structure was found as a homotetramer (Figure 5B). Since it is homotetramer composition, the chain A was kept for the docking and the rest of the other chains were removed. The literature helped to assign following active site residues (Radaev et al., 2000): LYS55, LYS60, ARG63, SER64, ALA116, LYS138, ARG168, HIS202, and GLU239. Figure 6 shows the active site regions of the protein 1D9E.

**Figure 7:**
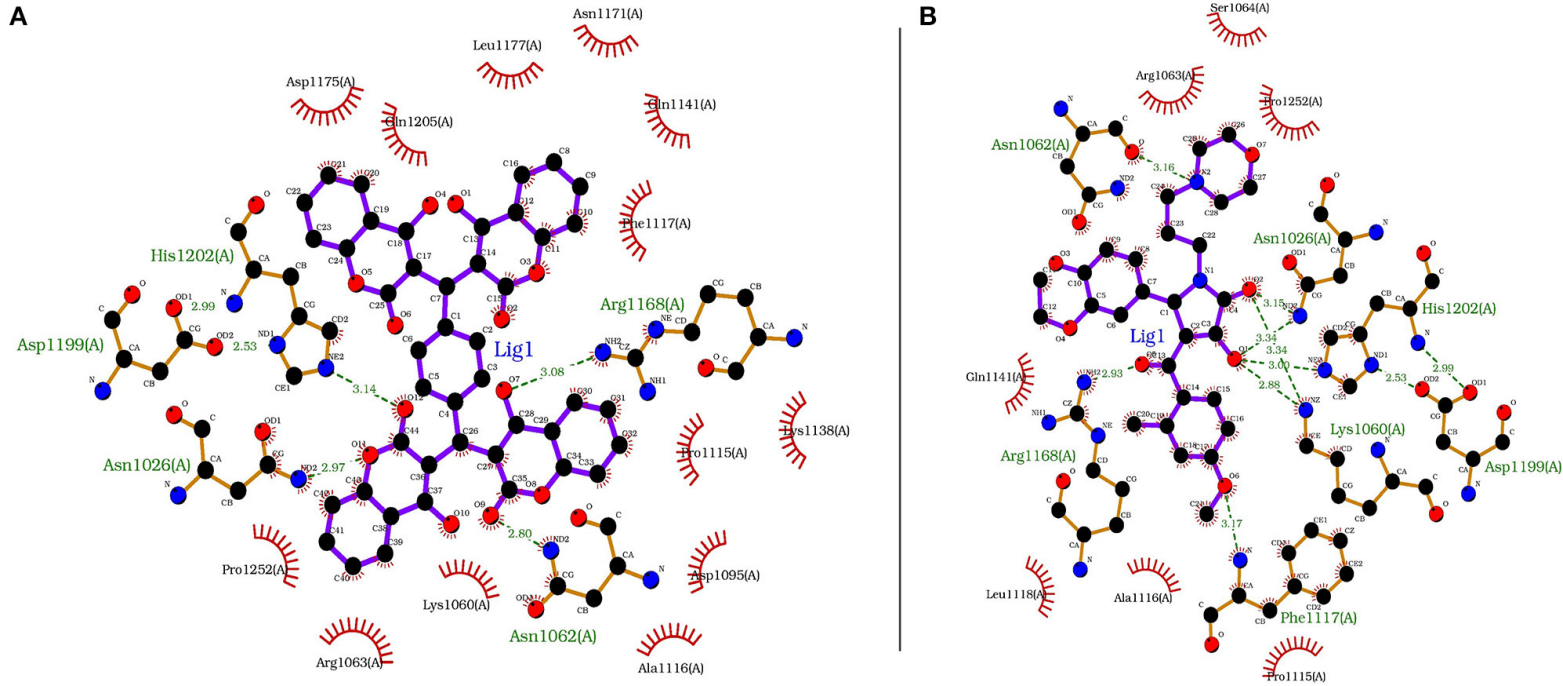
The receptor (PDB code: 1D9E) interacting with **(A)** ZINC95543764 (−11.6 Kcal/mol) and **(B)** ZINC20057784 (−7.0 Kcal/mol).

Each compound from the ZINC database was docked using the AutoDock Vina tool to identify potential inhibitors of the enzyme. The corresponding energy score of each compound was estimated and retrieved for the purpose of ranking the compounds. A histogram was generated showing the frequency of compounds with respect to the scoring range. The histogram had a normal-like distribution, and showed that the maximum number of compounds were found in the scoring bin of −7.0 to −6.8 Kcal/mol. Since it is interesting to see how these compounds interact with the receptor, the compounds in this range were extracted using shell script. Top 5% compounds were selected from the histogram and prioritized as potential inhibitors of KDO8P synthase (Supplementary Dataset 5). To make a comparison, the 2D interactions of the top most energy scored (i.e., −11.6 Kcal/mol) and the most frequent scored (i.e., −7.0 Kcal/mol) compounds are presented in Figures 7A,B, respectively. We used LigPlot to generate the 2D interaction diagrams (Wallace et al., 1995). Table 2 has 2D structures of the compounds ZINC95543764 and ZINC20057784. Figure 7A shows that the compound ZINC95543764 mediates significant interactions with the active site and the residues in the vicinity. The main hydrogen bonding interactions include ARG1168, ASN1062, HIS1202, ASP1199, and ASN1026. This compound is a derivative of coumarin, and it is reported to have too low acute toxicity in mice, with high antimicrobial and antioxidant potential (Hamdi et al., 2008). Bactericidal and fungicidal efficacy of the naturally occurring or synthetic coumarin derivatives are commonly investigated in literature (Hamdi et al., 2008; Siddiqui et al., 2011; Dastan et al., 2016; Singh et al., 2017; Tan et al., 2017). To date, the significant inhibitory activity of several biologically active coumarin-based compounds has been demonstrated on *K. pneumoniae* (Siddiqui et al., 2011; Tan et al., 2017). Based on the antibacterial potential of the coumarin derivatives, we suggest ZINC95543764, the top most energy scored compound in this study, as a potential *Klebsiella* inhibitor. Figure 7B shows the interactions mediated between the compound ZINC20057784 and the active site residues of the receptor. The key hydrogen bonding interactions in this case are ASP1199, HIS1202, LYS1060, ASN1026, ASN1062, ARG1168, and PHE1117. The list of identified compounds can be tested in follow-up experimental studies.

**Table 2:** 2D structures of the compounds ZINC95543764 and ZINC20057784.

**ZINC ID**	**Name**	**Structure**	**Vina dock score**
ZINC20057784	(5R)-5-(3-Ethoxy-4-hydroxyphenyl)-4-[hydroxy-(4-methoxy-2-methylphenyl)methylidene]-1-(3-morpholin-4-ylpropyl)pyrrolidine-2,3-dione	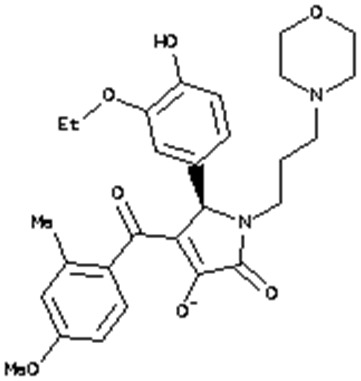	−7.0 Kcal/mol
ZINC95543764	3-[[4-[Bis(4-hydroxy-2-oxochromen-3-yl)methyl]phenyl]-(4-hydroxy-2-oxochromen-3-yl)methyl]-4-hydroxychromen-2-one	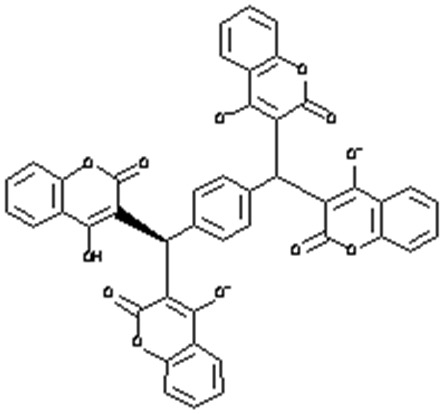	−11.6 Kcal/mol

### Metabolite-Centric Approach

The putative drug targets identified through the gene-centric approach were extended via the metabolic-centric approach, which is based on FBA-based prediction of EMs. The metabolic-centric approach follows similar steps to the gene-centric approach. First, EMs are predicted using FBA based metabolite-blocking simulations. Then, among the identified EMs, those also available in human cells and those associated with human homologous enzymes are filtered out. The structural analogs of remaining EMs are proposed as potential drug candidates. The enzymes associated with the outgoing reactions of these EMs are potential drug targets, whose simultaneous blocking will cause cell death. The metabolite-centric approach enables identification of sets of novel drug targets that could not be predicted by the gene-centric approach. The steps of the metabolic-centric approach are detailed below.

Firstly, EMs were determined by constraining the flux through the associated outgoing reaction(s) of each metabolite in the model to zero. In this manner, the effect of the absence of a metabolite on the growth rate of the organism was simulated. Forty-one EMs were predicted via the SM simulation. Of these, 23 metabolites were found to be essential for the bacterial growth in the HBF. The generic host medium simulation led to identification of the same EM list as HBF simulation. These metabolites were filtered to eliminate the compounds also involved in the human metabolism. Removal of the common metabolites is crucial to decrease the risk of drug-related side effects. In this regard, the metabolites found in both *K. pneumoniae* metabolism and human metabolism were screened using the list of 807 common metabolites given in Supplementary Dataset 6. Seventeen out of forty-one EMs from the SM simulation and 5 out of 23 EMs from the HBF (or the generic host medium) simulation were listed as pathogen-specific compounds while the remaining EMs were excluded. To explore the putative drug targets, the enzymes catalyzing the reactions associated with the selected metabolites were analyzed in terms of homology to human enzymes. The EMs associated with homologous enzymes were excluded. A total of six enzymes (encoded by *ribC, ribH, mrcA, mrcB, pbpC*, and *fabI*) are associated with the remaining EMs, involved in the corresponding outgoing reactions (Table 3). Therefore, they were suggested as putative drug targets. The associated six metabolites are 4r5au [4-(1-D-Ribitylamino)-5-aminouracil], dmlz [6,7-Dimethyl-8-(1-D-ribityl)lumazine], uaagmda [undecaprenyl-diphospho-N-acetylmuramoyl-(N-acetylglucosamine)-L-ala-D-glu-meso-2,6-diaminopimeloyl-D-ala-D-ala], t3c5ddeceACP (trans-3-cis-5-dodecenoyl-ACP), t3c7mrseACP (trans-3-cis-7-myristoleoyl-ACP), and t3c9palmeACP (trans-3-cis-9-palmitoleoyl-ACP). They were proposed to be drug analogs. This is because drugs share structural similarities with metabolites. Use of such drugs is equivalent to simultaneous suppression of all outgoing reactions around each EM. This ensures that inhibitors (structural analogs of the metabolites) primarily target synthetic lethal interactions, enabling more efficient treatments. The predicted enzymes [riboflavin synthase subunit α (RibC), riboflavin synthase subunit β (RibH), penicillin-binding protein 1A-C (PBP 1A, PBP 1B, and PBP 1C encoded by *mrcA, mrcB*, and *pbpC* genes, respectively), and enoyl-(acyl carrier protein) reductase (FabI)] are involved in several outgoing reactions, which are associated with the riboflavin, peptidoglycan, or fatty acid metabolism. Of these enzymes, RibC, RibH, and FabI were found to be independently essential, and the PBP 1A-C enzymes were identified as synthetic lethal. Characteristics of the final EMs and the associated genes are summarized in Table 3.

**Table 3:** List of the filtered essential metabolites and the associated putative drug targets as well as the outgoing reactions.

**Metabolite**		**Putative drug target (Enzyme)**					**Reaction**	
**Name**	**Metabolism**	**Name**	**Essential**	**Druggable**	**Broad- spectrum**	**Localization**	**Name**	**Essential**
4r5au	Riboflavin Synthesis	*ribC*	+	+	+	C	RBFSa	+
dmlz	Riboflavin Synthesis	*ribH*	+	+	+	C	RBFSb	+
uaagmda	Peptidoglycan Synthesis	*mrcA* *mrcB* *pbpC*	– – –	+ + +	+ + +	IM IM IM	MPTG/MPTG2	+/−
t3c5ddeceACP t3c7mrseACP t3c9palmeACP	Fatty Acid Synthesis	fabI^*^	+	+	+	IM	EAR161x_/_y EAR141x_/_y EAR121x_/_y	– – –

*C, cytoplasm; IM, inner membrane*.

* *Predicted by only sputum-macrophage (SM) simulation*.

Bacterial fatty acid synthesis is an essential process supporting formation of the cell membrane. Even if each step in this pathway is vital for the bacteria, FabI has received a particular interest owing to its rate-controlling role in the fatty acid synthesis. It catalyzes the last step in the elongation cycle of fatty acid synthesis including NAD(P)H dependent reduction of the trans-2-enoyl-ACP to acyl-ACP (Yao and Rock, 2016). There is an intense effort to develop inhibitors for this regulatory protein (Yao and Rock, 2016; Mistry et al., 2017). However, various resistance mutations in this enzyme render the available inhibitors ineffective (Yao and Rock, 2016). To address this challenge, novel inhibitors must be introduced. To this aim, the EMs identified in the current study may help researchers in the structure-based drug design.

RibC and RibH (encoded by *ribC* and *ribH*) were predicted using both gene- and metabolic-centric approaches. They are essential in riboflavin (vitamin B2) synthesis. Riboflavin is an essential precursor of FMN and FAD. Its synthesis is essential in the gram-negative bacteria lacking a transport system for riboflavin uptake while the *ribC* is conditionally essential in the bacteria with a riboflavin transporter. Disruption of the *ribC* was reported as lethal in the mutant *H. influenzae* cells cultured in a riboflavin-deficient medium (Saeed-kothe et al., 2004). Other targets including PBP 1A, PBP 1B, and PBP 1C participate in the peptidoglycan biosynthesis (Figure 4). Peptidoglycan is a cross-linked polymer in the periplasm that plays a critical role in the protection of the bacteria from rupturing by the high intracellular pressure (Vollmer and Höltje, 2000; Vollmer and Bertsche, 2008). The backbone of this mesh-like polymer consists of disaccharide peptide moiety of lipid II cross-linked by peptide bridges. Penicillin-binding proteins (PBPs) carrying transpeptidase and/or glycosyltransferase activities are involved in the final stages of peptidoglycan synthesis. Polymerization of peptidyl disaccharide subunits is managed through the glycosyltransferase activity, and cross-linking by peptide bridges is catalyzed through the transpeptidase activity (Derouaux et al., 2013; Mesleh et al., 2016). Inhibition of the peptidoglycan synthesis was reported to result in bacterial cell lysis and subsequently death of the cell (Derouaux et al., 2013). Here, the metabolite-centric approach supported this phenomenon by predicting the related enzymes as the putative targets. Furthermore, these enzymes were found as synthetic lethal. Prediction of such targets without the requirement of time-consuming multiple gene deletions is a prominent superiority of the metabolite-centric approach. Investigation of a drug mimicking the structure of uaagmda is a dramatically reasonable approach to narrow down the available chemical library compounds. Therefore, uaagmda analogs may be screened for the simultaneous suppression of PBP 1A-C. Such drugs may be more effective to diminish rapid resistance development when compared to single-target inhibitors.

## Conclusion

Genome-scale metabolic networks of different *K. pneumoniae* strains have been developed so far (Liao et al., 2011; Henry et al., 2017; Ramos et al., 2018; Norsigian et al., 2019), but to our knowledge they were not used for drug target discovery via the constraint-based analysis coupled to bioinformatic prioritization steps. This prompted us to investigate candidate drug targets for *K. pneumoniae* comprehensively via a network-based metabolism-centered approach. We identified the *K. pneumoniae* enzymes that are crucially involved in the bacterial survival. In all simulations, different host-mimicking environments and an improved biomass formation reaction were included in the GMN. Upon gene-centric approach, KdsA enzyme was found to be the high-ranked putative target highly satisfying most of the target prioritization criteria. This result encouraged us to investigate the potential KdsA inhibitors. We identified a list of compounds including ZINC95543764 and ZINC20057784, which can efficiently bind to the active site of the enzyme. It is important to note that a scarce number of KdsA inhibitors have so far been reported (Du et al., 1999; Birck et al., 2000; Grison et al., 2005; Le Calvez et al., 2009; Harrison et al., 2012; Smyth and Marchant, 2013). We provided an insight on the molecular nature of binding interactions. This may open new avenues to explore the novel inhibitors. We also presented additional promising putative targets and corresponding drugs, laying the foundation for future studies in the scope of this work (Supplementary Datasets 3, 4). In addition to the gene-centric approach, we employed the metabolite-centric approach. This yielded the synthetic lethal targets that could not be detected through the gene-centric approach. Collectively, the comprehensive effort on the investigation of the *K. pneumoniae* metabolism has enabled better understanding of the pathogenic phenotype and elucidation of the putative targets. Future studies can provide more evidence for the targets and inhibitors suggested in this study.

## Data Availability Statement

The raw data supporting the conclusions of this manuscript will be made available by the authors, without undue reservation, to any qualified researcher.

## Author Contributions

MC performed metabolism-based simulations and analyses. BS employed structure-based analyses. SD, RU, TÇ conceived and designed the study.

### Conflict of Interest

The authors declare that the research was conducted in the absence of any commercial or financial relationships that could be construed as a potential conflict of interest.
